# Screening and Application of DNA Markers for Novel Quality Consistency Evaluation in *Panax ginseng*

**DOI:** 10.3390/ijms26062701

**Published:** 2025-03-17

**Authors:** Siyuan Cai, Xuejiao Liao, Yidan Xi, Yang Chu, Shuang Liu, Hang Su, Deqiang Dou, Jiang Xu, Shuiming Xiao

**Affiliations:** 1 State Key Laboratory for Quality Ensurance and Sustainable Use of Dao-Di Herbs, Institute of Chinese Materia Medica, China Academy of Chinese Medical Sciences, Beijing 100700, China; wddcccsy@163.com (S.C.); xuejiaoliao1226@163.com (X.L.); xy_idan@163.com (Y.X.); ychu@icmm.ac.cn (Y.C.); 2 College of Pharmacy, Liaoning University of Traditional Chinese Medicine, Dalian 116600, China; deqiangdou@126.com; 3 Shanxi Institute of Functional Foods, Shanxi Agricultural University, Taiyuan 030031, China; shuangliu@sxau.edu.cn; 4 Northeast Asia Research Institute of Traditional Chinese Medicine, Key Laboratory of Active Substances and Biological Mechanisms of Ginseng Efficacy, Ministry of Education, Jilin Provincial Key Laboratory of Bio-Macromolecules of Chinese Medicine, Changchun University of Chinese Medicine, Changchun 130117, China; suhang0720@live.cn

**Keywords:** *Panax ginseng*, genetic polymorphism, quality consistency, HWEP

## Abstract

Quality control remains a challenge in traditional Chinese medicine (TCM). This study introduced a novel genetic-based quality control method for TCM. Genetic variations in ginseng were evaluated across whole-genome, chloroplast genome, and ITS2 DNA barcode dimensions. Significant genetic variations were found in whole-genome comparison, leading to the use of inter-simple sequence repeat markers to assess the genetic diversity of ginseng decoction pieces (PG), garden ginseng (GG), and ginseng under forest (FG). Fingerprints of ginseng samples revealed instability within some batches. These evaluations were transformed into information entropy to calculate the size of Hardy–Weinberg equilibrium population (HWEP). FG had significantly higher genetic and chemical minimum HWEP than GG (*p* < 0.05). Notably, a significant positive correlation was observed between the minimum HWEP for genetics and for chemistry (*r* = 0.857, *p* = 0.014). Genetic polymorphism analysis of ginseng has the potential to evaluate chemical quality consistency, offering a new method to ensure quality consistency in TCM.

## 1. Introduction

Traditional Chinese medicine (TCM) is an indispensable component of medical and health resources in China, playing a crucial role in promoting human health. However, its quality control faces significant challenges due to the inherent complexity of herbal materials. Unlike chemically synthesized drugs, TCM herbs are natural products whose quality is influenced by multiple factors, including genetic variations (species, medicinal parts), environmental conditions (growth, harvest time), and post-harvest processing (storage, extraction). These variables can lead to batch-to-batch inconsistency in chemical composition and pharmacological efficacy [1,2].

Traditionally, quality control of TCM relies on the identification of traits based on seeing, feeling, smelling, and tasting, which is highly subjective and poorly reproducible. In recent years, electronic noses and electronic tongues have been increasingly applied to the quality evaluation of TCM, with more objective and accurate information [3]. Currently, the chemistry-based strategy is the mainstream strategy for evaluating the quality consistency of TCM, which mainly relies on the spectroscopic and chromatographic characterization of active ingredients or content determination of characteristic ingredients [2]. Based on the systematic study of chemical components of TCM, fingerprinting, a comprehensive and quantifiable identification method, is used to evaluate the consistency of TCM. It has been recognized as one of the most suitable technologies to control the quality of TCM for its potential to reflect integral characterization of herbal drugs [4]. However, this is a detection method for production results, which cannot monitor the fluctuation of the quality parameters of natural herbs in the production process in real time. Additionally, given the current difficulty in elucidating the active ingredients of TCM, it may be more feasible to ensure the quality consistency of TCM through the control of raw materials and production processes.

The safety and quality of raw materials from a medicinal plant depend significantly on its intrinsic and extrinsic factors. As the World Health Organization has defined, the intrinsic factors include genetic influences, and extrinsic factors include environmental conditions, collection methods, cultivation, harvesting, and storage [5]. To ensure the consistent quality of raw materials, guidelines such as Good Agricultural Practice and Good Agricultural and Collection Practice have been established, which have yielded results on minimizing variation in raw materials and achieving batch-to-batch consistency [2]. However, the issue of quality stability in TCM has remained unresolved. Little attention has been paid to the intrinsic quality control of TCM. Among the limited studies that have addressed the intrinsic quality control of TCMs, only a few have emphasized the utilization of DNA barcoding technology for the identification of TCM, which always serves a single sample as the representative of a farm, a location, or a species with the underlying assumption that the population analyzed are uniform or that the difference within a population is negligible [6]. The assumption has been proven doubtful. Several studies showed clear evidence of genetic diversity and chemical variation within a population [5,6,7,8]. Hence, it is imperative to conduct a more meticulous characterization of the genetic diversity of TCM raw materials through molecular biology techniques, in order to achieve comprehensive control over their quality and stability.

The advancement of sequencing technologies has facilitated the increasing application of second-generation sequencing and third-generation sequencing in genomic characterization of medicinal materials [9,10]. Comprehensive genomic analyses of multiple TCM species have been conducted by our research team, including *Panax ginseng* [11,12,13], *Bungarus multicinctus* [14] and *Artemisia annua* [15,16]. These investigations have provided a comprehensive understanding of the genetic background underlying medicinal biosynthesis pathways, particularly elucidating the molecular mechanisms of pharmaceutically active compound synthesis, thereby establishing a foundation for correlating genetic determinants with phytochemical profiles. Nevertheless, the cost-prohibitive nature of whole-genome sequencing necessitates the development of alternative molecular markers for efficient genetic evaluation. Building upon the *A. annua* genomic framework, Ding et al. [17] suggested that ITS2 haplotype analysis is ideal tool for *A. annua* strain identification and population genetic homogeneity assessment. This finding is in accord with previous researches for multi-source herbs, such as licorice seeds [18] and Ephedra Herba [19], substantiating its reliability and applicability. Additionally, chloroplast genome- and whole-genome-based sequencing would provide a better picture on the genetic makeup of a plant, while the limited availability of resources and exorbitant costs has made its use extremely limited so far. Cost-effective techniques, for instance, inter-simple sequence repeats (ISSR), use dominant markers for genetic characterization and phylogenetic analysis, as they are simple, reproducible, and considered adequate to provide information on the basic differences in genotypes.

In this study, a paradigm for research was established with ginseng (*Panax ginseng* C. A. Mey), a more representative bulk medicinal material. After the selection of proper DNA markers, the ISSR marker was chosen to characterize the genetic polymorphism of ginseng under forest (FG), garden ginseng (GG), and ginseng decoction pieces (PG) samples. The Ultra-Performance Liquid Chromatography (UPLC) method was utilized to determine the chemical constituents of ginseng. Subsequently, the genetic diversity and chromatographic fingerprint results were transformed into corresponding information entropy values. Mathematical models established by Wang et al. [20] were applied to calculate the Hardy-Weinberg equilibrium population (HWEP) values. A correlation was established between genetic polymorphism and chemical quality fluctuations, providing novel insights and methodologies from a genetic perspective for achieving closer control over quality consistency and stability in production and clinical medication administration.

## 2. Results

### 2.1. Screening of DNA Markers and Sequence Analysis

**Figure 1:**
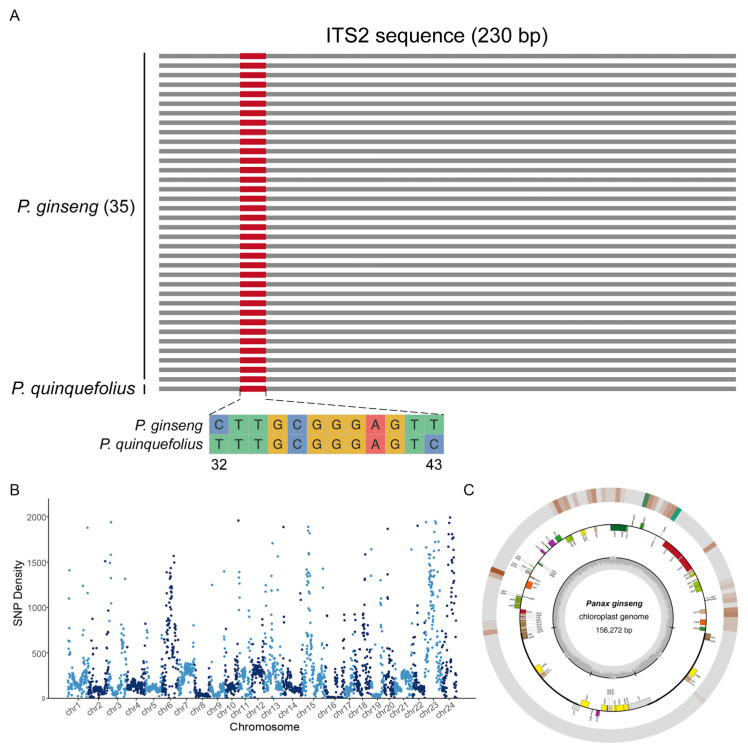
Genetic variation and SNP (single nucleotide polymorphism) density in *P. ginseng*. (**A**) ITS2 sequence comparison between *P. ginseng* and *P. quinquefolius*. ITS2 region shows high conservation among 35 ginseng samples and the interspecific nucleotide diversity in the ITS2 of *P. ginseng* and *P. quinquefolius* is represented by two SNPs (32 bp and 43 bp). (**B**) SNP density between the FG (ginseng under forest) and CG (garden ginseng) genome among 24 chromosomes. (**C**) Assembly of FG chloroplast genome (156,272 bp) and SNP density between FG and CG chloroplast genome, with 97.76% of SNPs concentrated in the LSC (large single copy) region. The outermost circle indicates SNP density, and SNP density increases progressively from brown to green, with green regions indicating higher SNP concentrations.

To explore genetic differences and the distribution patterns of ginseng, we conducted a comprehensive variation analysis across three genetic dimensions: the whole genome, the chloroplast genome, and the ITS2 DNA barcode. First, we analyzed the ITS2 sequences of 35 ginseng samples. Our analysis revealed that the ITS2 region was remarkably conserved across different ginseng samples, exhibiting minimal to no variation (Figure 1A). This high level of conservation highlights the stability of ITS2 as a genetic region among ginseng populations.

Our whole-genome analysis identified a total of 890,472 SNPs (single nucleotide polymorphisms) between FG and GG, demonstrating substantial genetic differentiation. Notably, these SNPs were unevenly distributed across chromosomes, with chromosome 24 exhibiting the highest level of variation. Specifically, chromosome 24 harbored approximately 117,743 variant sites, corresponding to an average of 776 variants per megabase (Figure 1B).

In parallel, a comprehensive comparative analysis of the chloroplast genomes between FG and GG was conducted. The chloroplast genome of FG was assembled with a size of 156,272 bp, while the genomic variations in GG were obtained based on alignment data. The comparative analysis revealed a relatively stable genetic structure, with the majority of observed differences concentrated within the large single-copy (LSC) region (Figure 1C). SNP distribution analysis across the chloroplast genome, using 10 kb bin intervals, identified a total of 134 variant sites, with 97.76% of these SNPs located in the LSC region. This concentration of variation within the LSC region highlights it as a potential hotspot for chloroplast genome diversity.

After the initial analysis of the genetic characteristics of Panax ginseng, the high conservation of the ITS2 region was observed across different samples, limiting its utility for assessing genetic diversity. Given the significant genetic differentiation revealed by whole-genome analysis, especially on chromosome 24, a more comprehensive and cost-effective molecular marker was needed to evaluate the genetic diversity of ginseng. Considering these factors, we selected ISSR markers as a practical and efficient tool for genetic diversity assessment due to their simplicity, low cost, and ability to provide basic differences in genotypes.

### 2.2. ISSR Analysis of Genetic Diversity in Ginseng

The ISSR analysis using eight primers on 21 ginseng samples generated a total of 92 amplified bands, of which 80 were polymorphic, yielding an average polymorphism rate of 87% (Table 1). The number of bands produced per primer ranged from 5 to 14, with an average of 11 bands per primer. Significant variations in amplification efficiency were observed among different primers, with UBC808 and UBC873 showing optimal performance (both achieving 100% polymorphism), while UBC809 exhibited a relatively lower polymorphism rate of 60%. These results demonstrate that the selected primers effectively revealed the genetic diversity of ginseng samples, providing reliable molecular markers for subsequent quality consistency research.

### 2.3. Genetic Similarity Coefficient and Cluster Analysis

**Figure 2:**
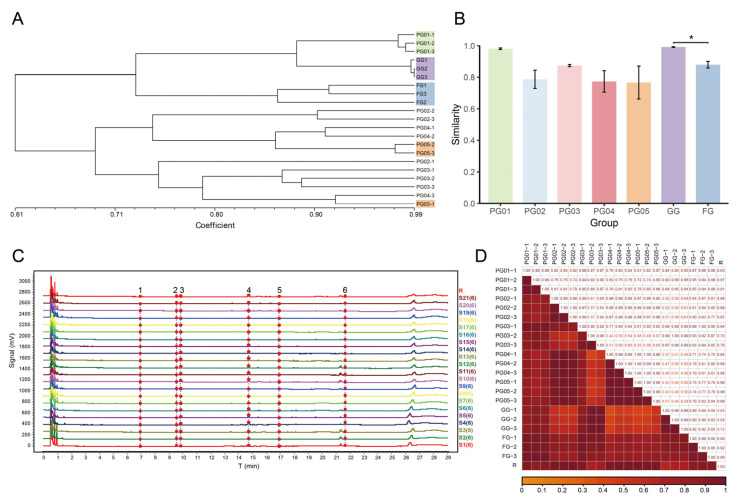
Genetic and chemical analysis of ginseng samples. (**A**) UPGMA cluster analysis of ginseng samples. (**B**) Comparison of genetic similarity coefficient among ginseng samples. (**C**) UPLC fingerprints after peak alignment, with six common peaks identified. (**D**) Heatmap of fingerprint similarity. * *p* < 0.05.

The genetic similarity coefficients among individuals were calculated using NTSYS-pc 2.10e, and a UPGMA (unweighted pair group method with arithmetic average) dendrogram was constructed among ginseng samples (Figure 2A). For PG, the admixture within each batch was characterized by the average of the pairwise similarity coefficients among three samples within that batch (Figure 2B). The calculated average genetic similarity coefficients for the five batches of PG ranged from 0.7667 to 0.9811, indicating varying degrees of genetic admixture among the batches. Specifically, PG05 exhibited the lowest average genetic similarity coefficient of 0.7667 ± 0.1809, indicating the highest degree of genetic admixture, which was reflected in the dendrogram where PG05-1 did not cluster with the other two samples of PG05. Conversely, PG01 had the highest similarity coefficient of 0.9811 ± 0.0084, suggesting a potentially more homogeneous source for this batch, as all three samples within this batch clustered together in the dendrogram. Additionally, the three samples of GG had an average similarity coefficient of 0.9918 ± 0.0036, indicating close genetic proximity among them. In contrast, the average similarity coefficient for FG ginseng was 0.8797 ± 0.0368, and a significant difference (*p* < 0.05) was observed between FG and GG, suggesting that despite originating from the same region, FG possesses a more complex genetic background.

### 2.4. Analysis of UPLC Fingerprint

#### 2.4.1. Method Validation

To validate the fingerprint analysis method, experiments were conducted to measure accuracy, repeatability, and stability (Table S1). Using peak 4 as the reference peak (S), the ginseng sample FG1 was prepared according to the method outlined in Section 4.5 to obtain the test solution. The test solution was then injected six times consecutively under the chromatographic conditions specified in Section 4.6. Calculations revealed that the relative standard deviation (RSD) of the relative retention time (RRT) for the common peaks was less than 0.24%, and the RSD of the relative peak areas (RPA) was less than 3.30%. Additionally, six parallel sample solutions of FG1 were injected and measured. The calculations showed that the RSD of the RRT for the common peaks was less than 0.30%, and the RSD of the RPA was less than 3.94%. Furthermore, the FG1 test solution was injected and measured at 0, 2, 4, 6, 8, 12, and 24 h after preparation. The calculations indicated that the RSD of the RRT for the common peaks was less than 0.17%, and the RSD of the RPA was less than 4.33%.

#### 2.4.2. UPLC Analysis and Similarity Evaluation

According to the detection method for the characteristic chromatogram of saponins, six common peaks were identified among all the test solutions of the ginseng samples at a wavelength of 203 nm, with three of these peaks identified, as shown in Figure 2C. The similarity among the samples was calculated using the Similarity Evaluation System for Chromatographic Fingerprinting of Traditional Chinese Medicine (2012 version), and the similarity values are listed in Table S2. Preliminary observation of the heatmap (Figure 2D) revealed that there were cases where the intra-batch similarity was below 0.9, indicating instability within the batches. The inter-batch similarity was even lower, particularly when comparing PG03 and GG with other batches. The average similarity values among the fingerprint chromatograms of the three test solutions for each of the five batches of ginseng slices were 0.990 ± 0.005, 0.993 ± 0.005, 0.934 ± 0.043, 0.996 ± 0.004, and 0.993 ± 0.006, respectively. The average similarities for GG and FG were 0.989 ± 0.007 and 0.963 ± 0.023, respectively. ANOVA (One-Way Analysis of Variance) testing showed that the similarity of the PG03 batch was significantly lower than that of the other batches (*p* < 0.05), combined with the highest RSD value, indicating the most prominent chemical fluctuation in this batch. Additionally, FG also exhibited relatively large chemical fluctuations.

### 2.5. The HWEP Study Based on the Information Entropy

#### 2.5.1. The Information Entropy and the MQS (Minimum Quantity for One Sampling) Determined by the ISSR Amplified Bands

**Figure 3:**
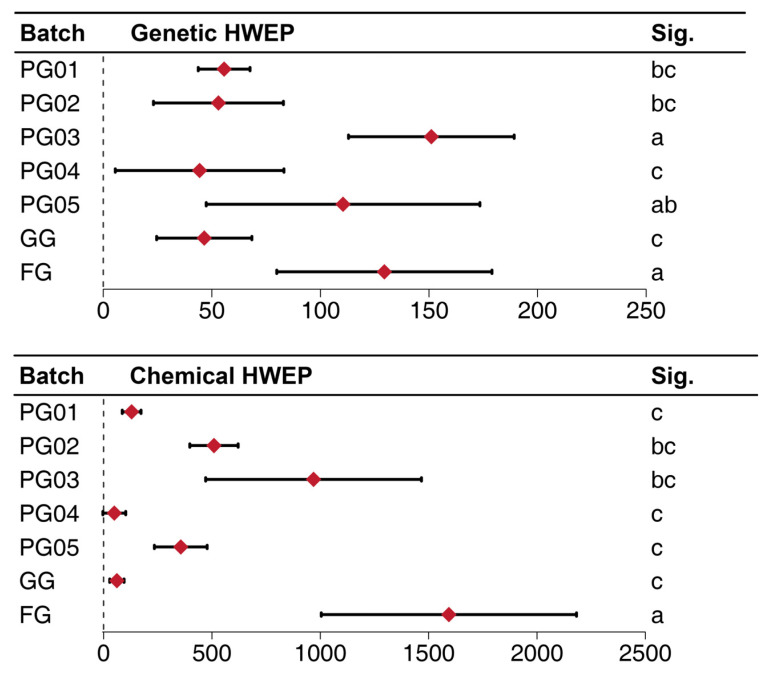
Comparison of HWEP (Hardy–Weinberg equilibrium population) of different ginseng samples. For both genetic data (means ± SEs, *n* = 8) and chemical data (means ± SEs, *n* = 6), different letters (a, b, c) indicate significant differences (*p* < 0.05, LSD-t test).

Based on Equations (1) and (2) outlined in Section 4.7, the HWEP size (*n*) within each batch could be calculated, as detailed in Table 2. By integrating the LOOCV (Leave-One-Out Cross-Validation) method proposed in Section 4.8, eight sets of HWEP sizes were obtained for each batch, with each equilibrium unit weighing 1.000 g, thereby determining the MQS. Further calculations of the mean and standard deviation among these eight sets of data are illustrated in Figure 3. The genetic HWEP sizes of the PG04 and GG batches were relatively lower compared to other batches, indicating a more homogeneous genetic background for these two batches. Conversely, the PG03 and FG batches exhibited higher HWEP sizes, suggesting a potentially more heterozygous genetic origin. A comparison between the FG and GG batches revealed a statistically significant difference in their genetic backgrounds (*p* = 0.001).

#### 2.5.2. The Information Entropy and the MQS Determined by Fingerprint Analysis

In analogy to the processing approach utilized for genetic outcomes, the statistical results of the fingerprints presented in Table 3 and Figure 3 were obtained by integrating Equations (3) and (5) from Section 4.7 with the LOOCV method proposed in Section 4.8. As evident from Figure 3, the chemical fluctuation of FG was significantly higher than that of the other six batches, while the chemical fluctuation of GG was comparable to the results of the other PG. Given that the three ginsenosides Rg_1_, Re, and Rb_1_ stipulated in ChP were still predominantly used as detection indicators during production, a calculation was also conducted considering only these three components as characteristic peaks, yielding the statistical results presented in Table 3.

**Figure 4:**
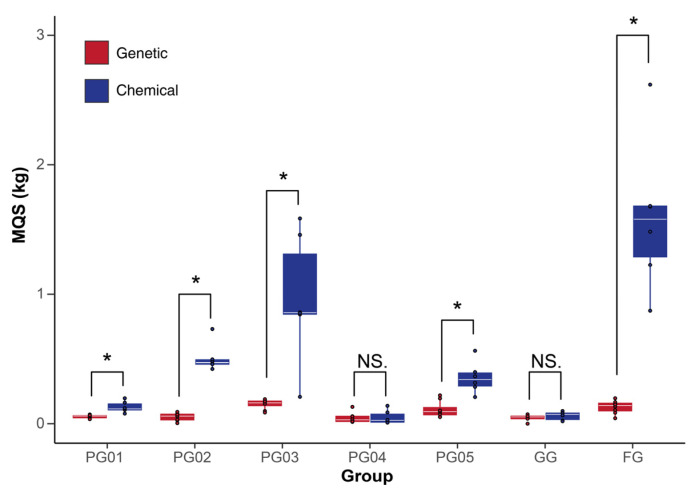
Comparison between genetic and chemical MQS within same batch of ginseng. *t*-test was used to test difference between genetic MQS data (means ± SEs, *n* = 8) and chemical MQS data (means ± SEs, *n* = 6). NS: not significant, * *p* < 0.05.

A comparison between the chemical MQS and the genetic MQS obtained from the same batches revealed that, except for PG04 and GG, the chemical MQS of the other five batches of ginseng samples were significantly higher than their respective genetic MQS (*p* < 0.05), as shown in Figure 4.

#### 2.5.3. Correlation Analysis Between Chemical Composition Fluctuations and Genetic Diversity

**Figure 5:**
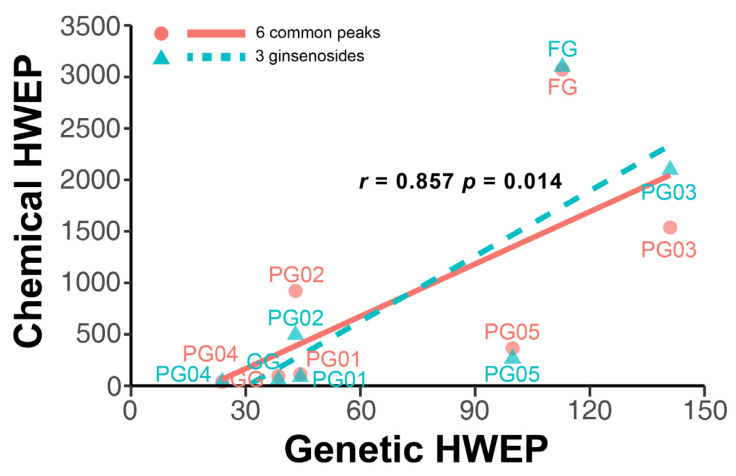
Correlation analysis between chemical compositions fluctuation and genetic diversity characterized by sic common peaks (marked in red) and three identified ginsenosides (marked in green). Spearman’s correlation analysis was conducted and significant positive correlation was found, with correlation coefficient of 0.857.

The HWEP size (*n*), derived from the entropy variation in genetic and chemical information within batches of ginseng, was employed as a parameter to quantify the genetic and chemical fluctuations within these batches, thereby investigating the correlation between the two. The chemical HWEP size was established based on either six characteristic peaks or three ginsenosides, with correlations established separately, as illustrated in Figure 5. Through Spearman’s correlation analysis, it was found that there was a significant positive correlation (*p* < 0.05) between the genetic HWEP size and the chemical HWEP size, with a correlation coefficient of 0.857. This indicated that the trends of genetic and chemical fluctuations within ginseng sample batches were consistent, suggesting that genetic heterogeneity could largely reflect the chemical fluctuations. Furthermore, similar results to those obtained using multiple characteristic peaks could be achieved through the analysis of three ginsenosides, which, in practical scenarios, offered greater convenience and operability.

## 3. Discussion

The production of TCM products constitutes an exceptionally intricate process, influenced by multiple factors, which results in poor quality consistency and ultimately leads to unstable clinical efficacy. Among these factors, raw materials represent the foremost contributor to quality inconsistency [21]. However, research on control measures for the internal genetic factors of raw materials remains relatively scant. This study, taking the bulk medicinal material ginseng as an example, selected PG, GG, and FG as representative samples. It focused on elucidating the correlation between the genetic polymorphism of medicinal materials and fluctuations in their chemical quality, thus providing a novel protocol for the quality control of TCM.

DNA barcoding, as a cost-effective, standardized, and rapid method for species identification, has significantly advanced the authentication of herbs. Liu et al. [18] pioneeringly introduced next-generation sequencing and single-molecule real-time sequencing technologies into the DNA barcoding of licorice, which has multiple botanical origins, successfully identifying the genetic diversity within licorice samples. This approach not only facilitated the identification of the botanical origins of licorice seeds but also unveiled the potential of DNA barcoding in assessing genetic diversity. Similar methodologies were replicated in Ephedra Herba [19], another herb with multiple botanical origins. DNA barcoding, leveraging relatively short and conserved genetic sequences for species identification, is simple to operate, accurate, and efficient. Moreover, its sequencing-based nature ensures high reproducibility and independence from specific instruments [22], prompting us to prioritize it as a tool for genetic diversity assessment in this study. However, ginseng samples exhibited no variation in the ITS2 region, aligning with findings of Kim et al. [23] that different strains of *Panax ginseng* showed no variation in ITS2. Ma et al. [24] reported intraspecific variation in the ITS2 sequence of Panax ginseng, but the variant strains originated from Hubei, which is not a major ginseng-producing area, suggesting that these results might be coincidental. In summary, the ITS2 region appeared stable within ginseng species and may not be suitable as a tool for genetic diversity assessment. Subsequently, we examined variations in the chloroplast genome and the whole-genome. Variations in the chloroplast genome were still scarce, whereas more variations were detectable across the whole-genome. However, whole-genome sequencing is costly. To obtain genome-wide fingerprints at a lower cost, this study ultimately employed ISSR molecular markers as indicators for genetic diversity assessment.

Various types of ginseng samples, including PG, GG, and FG, were collected for investigation in this study. These three types of samples are utilized differently in the market. The cultivated ginseng samples, GG, originated from a relatively homogeneous source and exhibited the closest genetic relationship, which may be attributed to the long-term artificial selection and standardized cultivation protocols that have led to a narrowing of their genetic background. These findings align with previous reports of diversity loss in GG revealed by RAPD markers [25]. Consequently, the chemical variations among GG were minimal. However, even among FG samples from the same locality, significant genetic and chemical variations were observed. PG are the most commonly used form in clinical practice but are also more difficult to trace and control compared to raw medicinal materials. The analysis of genetic consistency using ISSR molecular markers revealed genetic admixture within batches of PG. This may indicate genetic admixture during ginseng cultivation or admixture of raw medicinal materials during the preparation of batches, which could further contribute to chemical variations. In summary, these findings suggest that to ensure the consistency of TCM product quality in the future, it is necessary to strengthen the breeding of TCM materials to stabilize the intrinsic influencing factor, especially for FG, laying the foundation for future breeding and clinical use. Secondly, genetic admixture should be minimized during production.

Although ISSR molecular markers and UPLC chromatographic analysis techniques have been widely used in ginseng research, this study is the first to introduce the HWEP model into the quality evaluation system of ginseng, innovatively establishing a quantitative correlation between genetic polymorphism and chemical fluctuations. This approach provides a scientific basis for ginseng quality control from a genetic perspective for the first time. Specifically, to quantify genetic and chemical variations, this study introduced the principles of information entropy and a mathematical model for determining MQS. Bands and chromatographic fingerprints were converted into information entropy, and the fluctuations in information entropy were then translated into the HWEP size. The value of *n* directly reflected the degree of fluctuation. Ultimately, a positive correlation between genetic and chemical variations was established. Furthermore, the MQS calculated could guide production process. It is noteworthy that the chemical HWEP size obtained from chromatographic fingerprint was often larger than the corresponding genetic HWEP size for the same batch, and the corresponding MQS was also typically significantly higher than that obtained genetically. This finding shares some similarities with the results obtained by Wang et al. [20] on *Houttuynia cordata* and may be related to the quantitative nature of chromatographic fingerprint. From an information perspective, chromatographic fingerprint may more accurately reflect the true value of the equilibrium population. However, genetic analysis is simpler and more convenient. If the MQS obtained through genetic analysis can control the variation within an acceptable range within batches, it can reduce the cost of practical applications such as sampling inspection and possess greater potential for promotion. To accurately reflect the HWEP, it is essential not only to identify the optimal expression forms that can precisely characterize the polymorphism of TCM but also to develop extraction and formulation processes based on the MQS followed by clinical validation of its stability. Only the MQS that has undergone rigorous clinical validation and can ensure stable therapeutic efficacy can truly achieve quality control of TCM. In summary, this study provides new tools for the science of TCM regulation, addressing fluctuations in quality control starting from raw materials. It complements existing regulations that can only test production outcomes and promotes the development of quality consistency control towards the “Quality by Design” phase.

## 4. Materials and Methods

### 4.1. Sample Collection and Reagents

Thirty-five ginseng samples (Table S3) were selected for ITS2 sequence analysis. Then, a total of 21 ginseng samples were utilized, which contained 3 randomly selected slices taken from each of the 5 batches of PG, amounting to 15 samples. Additionally, three samples were obtained from FG and three from GG, respectively. Detailed information is presented in Table 4.

Ginsenoside Rg_1_ (Lot number CFS202301, purity > 98%) was purchased from Wuhan ChemFaces Biochemical Co., Ltd. (Wuhan, China), ginsenoside Re (Lot number 110754–202129, purity > 96%) was purchased from National Institute for Food and Drug Control (Beijing, China), and ginsenoside Rb_1_ (Lot number Yx122821, purity > 98%) was purchased from Nanjing Plant Origin Biological Technology Co., Ltd. (Nanjing, China). HPLC-grade methanol and acetonitrile were purchased from Thermo Fisher Scientific (Waltham, MA, USA). All other reagents and chemicals used in this study were of analytical grade.

### 4.2. ITS2 Sequences Analysis

DNA was extracted from fresh leaves of 35 ginseng samples listed in Table S3, which were pulverized in liquid nitrogen. The Plant Genomic DNA Kit (Tiangen Biotech Co., Ltd., Beijing, China) was used and DNA was stored at −20 °C. The ITS2 sequences were obtained in a reaction mixture containing 1 × Rapid Taq Master Mix (Vazyme Biotech Co., Ltd., Nanjing, China), 1 μM of each primer (forward: 5′-YGACTCTCGGCAACGGATA-3′; reverse: 5′-RGTTTCTTTTCCTCCGCTTA-3′), and ~100 ng DNA templates. The PCR amplification conditions consisted of predenaturation at 94 °C for 5 min, 30 cycles of amplification (94 °C for 1 min, 55 °C for 1 min, and 72 °C for 1.5 min), and a final extension at 72 °C for 7 min. The ITS2 amplicon was sequenced by Illumina Miseq PE300 (San Diego, CA, USA) at a depth of no less than 30 thousand pieces per sample. Sequencing service was provided by Personal Biotechnology Co., Ltd., Shanghai, China. All sequencing results are quality controlled. The sample sequences of ITS2 amplicons of 35 ginseng samples were aligned by the Burrows–Wheeler Aligner-Minimum Exact Match (BWA-MEM) (v 0.7.17).

### 4.3. ISSR Analysis

DNA was extracted using the same method as Section 4.2, from a total of 21 dried ginseng decoction pieces, which contained three randomly selected slices taken from each of five batches of PG, amounting to 15 samples, and three samples obtained from FG and three from GG, listed in Table 4. Then for ISSR, based on a literature review, we initially selected 10 primers for experimental analysis [26,27,28,29,30]. Through primer validation experiments, we identified that two primers exhibited issues such as fuzzy amplification bands and poor reproducibility (Figure S1). Consequently, we selected 8 primers demonstrating stable amplification, clear banding patterns, and good polymorphism for subsequent ISSR analysis (Table 1). To evaluate the reliability of the experimental method, we performed three independent parallel amplifications of the GG sample (Figure S2). The results demonstrated clear and consistent banding patterns across all three replicates, indicating that the PCR amplification method employed in this study exhibits excellent reproducibility and stability, providing reliable data for subsequent analyses. PCR was carried out in a 20 μL reaction mixture containing 10 μL of 2 × Rapid Taq Master Mix, 0.5 μM of each primer, and ~40 ng DNA templates. The amplification was performed under the following conditions: predenaturation at 94 °C for 5 min, 45 cycles of denaturation at 94 °C for 40 s, annealing at 50 °C for 40 s, and extension at 72 °C for 1 min, then extension at 72 °C for 7 min. PCR products were separated in a 2% agarose gel. Amplified band patterns were scored as “1” when the band was present and “0” when the band was absent. Based on the distinguished results, a binary data matrix was obtained. Genetic similarity was calculated and a dendrogram was constructed according to UPGMA of NTSYS-pc 2.10e.

### 4.4. Genome Sequencing and SNP Calling

The DNA samples from FG1 and GG1 were sequenced using the Illumina platform. Paired-end (PE150) libraries with an average insert size of 150 bp were constructed following the manufacturer’s protocol provided by Illumina (San Diego, CA, USA). The raw sequencing data were quality-controlled using Trimmomatic software (v0.39) with the following parameters, HEADCROP:5, MINLEN:18, TRAILING:30, and AVGQUAL:20, ensuring that the filtered reads met the requirements for subsequent analyses.

The Illumina sequencing reads from FG1 were aligned to the GG1 genome using BWA with the BWA-MEM algorithm. These alignments were subsequently used for variant calling with DeepVariant (v1.6.0). Low-quality variants were filtered out based on the following criteria: minimum read depth (minDP) > 3 and quality score > 30. The filtered SNP data were used to analyze the distribution and density of SNPs across different chromosomes. Next, the filtered reads were aligned to the published chloroplast reference genome (KC686331.1) using BWA-MEM. Similarly to the genome-wide SNP calling, DeepVariant was employed to perform deep learning-based variant calling. The distribution of variants across different chloroplast regions, including the LSC, small single-copy (SSC), and inverted repeat (IR) regions, was analyzed and summarized.

### 4.5. Preparation of Samples for UPLC Analysis

Approximately 1.000 g of PG was accurately weighed and placed in 30 mL of water for 30 min and then refluxed for 60 min. Then, the decoction was filtered and the residue was refluxed for 40 min in 20 mL of added water. The filtrates were combined and adjusted to 50 mL using water. To prepare the solution, 800 μL of the decoction was mixed with 800 μL of methanol, centrifuged at 14,000 r/min for 5 min, and then filtered through a 0.22 μm nylon filter before injection.

### 4.6. UPLC Analysis

The samples were analyzed using a Waters ACQUITY UPLC H-Class system equipped with a Waters (Milford, MA, USA) ACQUITY UPLC^®^ BEH C18 column (2.1 mm × 100 mm, 1.7 μm) at 30 °C, the injection volume was 2 μL and the flow rate was set at 0.4 mL/min. The mobile phase consisted of 0.1% phosphoric acid water (mobile phase A) and acetonitrile (mobile phase B). The gradient program was as follows: 0–4 min, the proportion of phase A goes from 89 to 79%; 4–7 min, hold 79% A; 7–12 min, from 79 to 68% A; 12–16 min, hold 68% A; 16–17 min, from 68 to 55% A; 17–20 min, from 55 to 30% A; 20–21 min, from 30 to 10% A; 21–23 min, from 10 to 5% A.

### 4.7. HWEP Evaluation Based on ISSR and Chromatographic Fingerprint

To assess the variability within and between the batches of PG, as well as to determine the sample size required to equilibrate such variability, a mathematical model based on the principle of information entropy for determining the MQS size, as proposed by Wang et al. [20], is referenced. For ISSR amplification bands, the following calculation can be used:

(1) $$H=-Cln\frac{m_{2}}{m_{1}},$$

Here, *H* represents the information entropy, *C* is a constant, *m*_1_ represents the total number of bands that would potentially appear after amplification with all primers for a given sample, and *m*_2_ represents the actual number of bands obtained through amplification for that sample. Based on this, the size of HWEP can be calculated as follows:

(2) $$n\geq 100{\left(t_{\alpha ,\;n-1}+u_{\beta }\right)}^{2}{RSD}^{2},$$

Here, *n* serves as the size of HWEP. Specifically, when the significance levels for both type I and type II errors are set at *α* = 0.05 and *β* = 0.05, respectively, $\left(t_{\alpha ,n-1}+u_{\beta }\right)$ = 10.8. Using this equation, the value of *n* can be determined.

Regarding the calculation of the information content in fingerprint chromatography, the formula is as follows:

(3) $$S_{p}=\sum_{i=1}^{n}p_{i1}lnp_{i2},$$

where *S_p_* represents the total information entropy of the chromatographic fingerprint, *p_i_*_1_ represents the response value (i.e., peak area) of each characteristic peak in the chromatographic fingerprint of an individual herbal medicine from a genetically balanced population, and *p_i_*_2_ represents the percentage of the response value of the corresponding characteristic peak in the entire fingerprint.

The information entropy expressed by each individual’s fingerprint profile is plotted on the *x*-axis, and the frequency of occurrence of this information entropy is plotted on the *y*-axis, resulting in a normal distribution curve of information entropy frequency. Based on the normal distribution curve of information entropy probability, the HWEP size is calculated using statistical principles as follows:

(4) $$\frac{\left|S_{pi}-\bar{S_{p}}\right|}{\frac{\sigma }{\sqrt{n}}}\geq t_{0.01,\;n-1},$$

Here, $\bar{S_{p}}$ represents the mean of information entropy for the TCM material, *σ* represents the variance, *t* is the critical value at a confidence coefficient of 0.01, and *n* stands for the unit of medicinal plants.

When $t_{0.01/2}$ = 2.56, and $\left|S_{pi}-\bar{S_{p}}\right|\leq 10\%\bar{S_{p}}$, that is, at a confidence coefficient of 0.1, and when the quality control is within the specified content limits of the validity period (according to the regulations on the validity period of preparations, the difference in drug component changes should not be less than 10% of the original value, at which point the quality is considered stable), Equation (4) can be transformed into Equation (5). The value of the balanced population is as follows:

(5) $$n\geq {\left(K\cdot RSD\right)}^{2}$$

where *K* is the formulation control coefficient, with a value of *K* = 25.6. Thus, the size of HWEP can be calculated.

### 4.8. Data Analysis

The genetic polymorphism profiles and phytochemical fingerprinting data were subjected to LOOCV analysis, which operates by iteratively designating a single observation as the validation subset while utilizing the remaining *N* − 1 observations for model training [31]. This procedure was repeated *N* times until all observations had served as validation instances. In this study, for genetic analysis, ISSR amplification with *m* primers generated electrophoretic banding profiles representing allelic variations. To evaluate primer-specific bias, LOOCV was implemented by systematically excluding banding data from one primer per iteration (*m* total iterations). The retained *m* − 1 primer datasets were utilized to compute genetic diversity indices, generating m distinct subsets for subsequent population genetic analyses. For chemical consistency evaluation, an analogous LOOCV framework was applied to UPLC-derived chromatograms. Each iteration excluded one characteristic chromatographic peak from the fingerprinting dataset.

R-4.4.1 was used to perform ANOVA and correlation analysis.

## 5. Conclusions

In this study, we aimed to develop a new method to achieve better control over quality consistency in herbal products from a genetic perspective. First, we conducted a systematic genetic variation analysis on ginseng samples, which revealed notable differences in chromosomal dimension. Then, we chose ISSR marker as a cost-effective methodology to provide adequate information on the basic differences in genotypes. As chemical composition fluctuations within batches were characterized by UPLC chromatographic fingerprint, a correlation between genetic diversity and chemical fluctuation was preliminarily established, with MQS calculated for production. These findings have provided novel insights and methodologies from a genetic perspective for achieving closer control over quality consistency and stability in production and clinical medication administration.

## Figures and Tables

**Table 1:** ISSR (inter-simple sequence repeat) primers used for this study and number of polymorphic fragments.

Primer ID	Primer Sequence (5′-3′)	TNB	NPB	PPB (%)
UBC807	AGAGAGAGAGAGAGAGT	14	12	85.7
UBC808	AGAGAGAGAGAGAGAGC	12	12	100
UBC809	AGAGAGAGAGAGAGAGG	5	3	60.0
UBC823	TCTCTCTCTCTCTCTCC	13	12	92.3
UBC834	AGAGAGAGAGAGAGAGYT	13	10	76.9
UBC840	GAGAGAGAGAGAGAGAYT	10	9	90.0
UBC842	GAGAGAGAGAGAGAGAYG	11	8	72.7
UBC873	GACAGACAGACAGACA	14	14	100

TNB: the number of total bands; NPB: the number of polymorphic bands; PPB (%): the percentage of polymorphic bands.

**Table 2:** The information entropy and MQS (minimum quantity for one sampling) determined by the ISSR amplified bands of different samples.

	No.	TNB	ln m	H	RSD	*n*	MQS (kg)
PG01	PG01-1	70	−0.2733	0.3943	6.16%	44.30	0.044
	PG01-2	72	−0.2451	0.3536			
	PG01-3	70	−0.2733	0.3943			
PG02	PG02-1	33	−1.0253	1.4792	6.07%	43.02	0.043
	PG02-2	37	−0.9109	1.3141			
	PG02-3	34	−0.9954	1.4361			
PG03	PG03-1	47	−0.6716	0.9690	10.99%	141.00	0.141
	PG03-2	41	−0.8082	1.1660			
	PG03-3	47	−0.6716	0.9690			
PG04	PG04-1	37	−0.9109	1.3141	4.52%	23.86	0.024
	PG04-2	40	−0.8329	1.2016			
	PG04-3	38	−0.8842	1.2756			
PG05	PG05-1	36	−0.9383	1.3536	9.25%	99.80	0.998
	PG05-2	42	−0.7841	1.1312			
	PG05-3	40	−0.8329	1.2016			
GG	GG1	81	−0.1273	0.1837	5.75%	38.53	0.039
	GG2	82	−0.1151	0.1660			
	GG3	81	−0.1273	0.1837			
FG	FG1	50	−0.6098	0.8797	9.83%	112.79	0.113
	FG2	47	−0.6716	0.9690			
	FG3	53	−0.5515	0.7956			

TNB: the number of total amplification bands; m: m_2_/m_1_, m_1_ represents the total number of bands that would potentially appear after amplification with all primers for a given sample, and m_2_ represents the actual number of bands obtained through amplification for that sample; RSD: relative standard deviation; *n*: HWEP value; MQS: minimum quantity for one sampling.

**Table 3:** The information entropy and the MQS determined by fingerprint analysis and characterized by six common peaks and three identified ginsenosides.

No.	6 Common Peaks				3 Identified Ginsenosides			
	Information Entropy	RSD	*n*	MQS (kg)	Information Entropy	RSD	*n*	MQS (kg)
PG01	1.08 × 10^5^	9.85%	113.07	0.113	1.01 × 10^5^	8.54%	85.13	0.085
	9.26 × 10^4^				8.81 × 10^4^			
	9.09 × 10^4^				8.66 × 10^4^			
PG02	2.44 × 10^5^	28.10%	920.99	0.921	1.61 × 10^5^	20.62%	495.83	0.496
	2.05 × 10^5^				1.88 × 10^5^			
	1.36 × 10^5^				1.23 × 10^5^			
PG03	7.52 × 10^4^	36.31%	1537.46	1.537	7.39 × 10^4^	42.41%	2097.62	2.098
	4.51 × 10^4^				3.59 × 10^4^			
	3.92 × 10^4^				3.92 × 10^4^			
PG04	1.73 × 10^5^	5.66%	37.37	0.037	1.57 × 10^5^	5.79%	39.15	0.039
	1.57 × 10^5^				1.42 × 10^5^			
	1.57 × 10^5^				1.43 × 10^5^			
PG05	1.73 × 10^5^	17.66%	363.74	0.364	1.60 × 10^5^	15.08%	265.30	0.265
	1.39 × 10^5^				1.31 × 10^5^			
	1.23 × 10^5^				1.20 × 10^5^			
GG	2.54 × 10^4^	8.97%	93.85	0.094	1.84 × 10^4^	7.06%	58.17	0.058
	2.48 × 10^4^				2.09 × 10^4^			
	2.15 × 10^4^				1.88 × 10^4^			
FG	3.64 × 10^4^	51.30%	3070.03	3.070	3.33 × 10^4^	51.54%	3098.48	3.098
	3.52 × 10^4^				3.44 × 10^4^			
	8.09 × 10^4^				7.68 × 10^4^			

RSD: relative standard deviation; *n*: HWEP value; MQS: minimum quantity for one sampling.

**Table 4:** Information on ginseng samples for ISSR and UPLC analysis.

Sample	Type	Place of Origin
PG01	Decoction pieces	Market of Jilin Province, China
PG02	Decoction pieces	Market of Jilin Province, China
PG03	Decoction pieces	Market of Jilin Province, China
PG04	Decoction pieces	Market of Jilin Province, China
PG05	Decoction pieces	Market of Jilin Province, China
FG1	Ginseng under forest	Tonghua City, Jilin Province, China
FG2	Ginseng under forest	Tonghua City, Jilin Province, China
FG3	Ginseng under forest	Tonghua City, Jilin Province, China
GG1	Garden ginseng	Tonghua City, Jilin Province, China
GG2	Garden ginseng	Tonghua City, Jilin Province, China
GG3	Garden ginseng	Tonghua City, Jilin Province, China

## Data Availability

Data are contained within the article and Supplementary Materials.

## Supplementary Materials

The following supporting information can be downloaded at: https://www.mdpi.com/article/10.3390/ijms26062701/s1.
