# Supplementary material for: Screening and Application of DNA Markers for Novel Quality Consistency Evaluation in Panax ginseng
Source: Int J Mol Sci. 2025 Mar 17;26(6):2701. doi: 10.3390/ijms26062701 (PMC11942579; doi:10.3390/ijms26062701)
Supplement: Supplementary file 1 [file ijms-26-02701-s001.zip › Table S3. Information of ginseng samples for ITS2 sequence analysis.pdf]

Table S3. Information of ginseng samples for ITS2 sequence analysis.

Sample Number	Sampling Location	Longitude	Latitude
KD-1	Xialicha, Zhenjiang Town, Kuandian County, Dandong City, Liaoning Province, China	N40°47'22.032"	E125°24'23.928"
KD-2	Xialicha, Zhenjiang Town, Kuandian County, Dandong City, Liaoning Province, China	N40°47'22.032"	E125°24'23.928"
KD-3	Xialicha, Zhenjiang Town, Kuandian County, Dandong City, Liaoning Province, China	N40°47'22.032"	E125°24'23.928"
KD-4	Xialicha, Zhenjiang Town, Kuandian County, Dandong City, Liaoning Province, China	N40°47'22.032"	E125°24'23.928"
KD-5	Xialicha, Zhenjiang Town, Kuandian County, Dandong City, Liaoning Province, China	N40°47'22.032"	E125°24'23.928"
HR-1	Daojianling, Huanren County, Benxi City, Liaoning Province, China	N42°25'21.948"	E125°41'0.03"
HR-2	Daojianling, Huanren County, Benxi City, Liaoning Province, China	N42°25'21.948"	E125°41'0.03"
HR-3	Daojianling, Huanren County, Benxi City, Liaoning Province, China	N42°25'21.948"	E125°41'0.03"
HR-4	Daojianling, Huanren County, Benxi City, Liaoning Province, China	N42°25'21.948"	E125°41'0.03"
HR-5	Daojianling, Huanren County, Benxi City, Liaoning Province, China	N42°25'21.948"	E125°41'0.03"
HRN-1	Xiaonancha, Erpengzi Town, Huanren County, Benxi City, Liaoning Province, China	N41°11'29.172"	E125°35'12.096"
HRN-2	Xiaonancha, Erpengzi Town, Huanren County, Benxi City, Liaoning Province, China	N41°11'29.172"	E125°35'12.096"
HRN-3	Xiaonancha, Erpengzi Town, Huanren County, Benxi City, Liaoning Province, China	N41°11'29.172"	E125°35'12.096"
HRN-4	Xiaonancha, Erpengzi Town, Huanren County, Benxi City, Liaoning Province, China	N41°11'29.172"	E125°35'12.096"
HRN-5	Xiaonancha, Erpengzi Town, Huanren County, Benxi City, Liaoning Province, China	N41°11'29.172"	E125°35'12.096"
FX-1	Xincheng District, Fusong County, Baishan City, Jilin Province, China	N42°12'0.93"	E127°28'0.942"
FX-2	Xincheng District, Fusong County, Baishan City, Jilin Province, China	N42°12'0.93"	E127°28'0.942"
FX-3	Xincheng District, Fusong County, Baishan City, Jilin Province, China	N42°12'0.93"	E127°28'0.942"
FX-4	Xincheng District, Fusong County, Baishan City, Jilin Province, China	N42°12'0.93"	E127°28'0.942"
FX-5	Xincheng District, Fusong County, Baishan City, Jilin Province, China	N42°12'0.93"	E127°28'0.942"

Sample Number	Sampling Location	Longitude	Latitude
TH-1	Kuaidamao Town, Tonghua County, Tonghua City, Jilin Province, China	N41°40′28″	E125°45′22″
TH-2	Kuaidamao Town, Tonghua County, Tonghua City, Jilin Province, China	N41°40′28″	E125°45′22″
TH-3	Kuaidamao Town, Tonghua County, Tonghua City, Jilin Province, China	N41°40′28″	E125°45′22″
TH-4	Kuaidamao Town, Tonghua County, Tonghua City, Jilin Province, China	N41°40′28″	E125°45′22″
TH-5	Kuaidamao Town, Tonghua County, Tonghua City, Jilin Province, China	N41°40′28″	E125°45′22″
JA-1	Xinkaihe Base, Liangshui Township, Ji'an, Tonghua City, Jilin Province, China	N41°26'29.586"	E125°55'24.288"
JA-2	Xinkaihe Base, Liangshui Township, Ji'an, Tonghua City, Jilin Province, China	N41°26'29.586"	E125°55'24.288"
JA-3	Xinkaihe Base, Liangshui Township, Ji'an, Tonghua City, Jilin Province, China	N41°26'29.586"	E125°55'24.288"
JA-4	Xinkaihe Base, Liangshui Township, Ji'an, Tonghua City, Jilin Province, China	N41°26'29.586"	E125°55'24.288"
JA-5	Xinkaihe Base, Liangshui Township, Ji'an, Tonghua City, Jilin Province, China	N41°26'29.586"	E125°55'24.288"
FC-1	Dongtang Town, Fengcheng City, Dandong City, Liaoning Province, China	N40°25'16.101"	E124°18'43.38"
FC-2	Dongtang Town, Fengcheng City, Dandong City, Liaoning Province, China	N40°25'16.101"	E124°18'43.38"
FC-3	Dongtang Town, Fengcheng City, Dandong City, Liaoning Province, China	N40°25'16.101"	E124°18'43.38"
FC-4	Dongtang Town, Fengcheng City, Dandong City, Liaoning Province, China	N40°25'16.101"	E124°18'43.38"
FC-5	Dongtang Town, Fengcheng City, Dandong City, Liaoning Province, China	N40°25'16.101"	E124°18'43.38"
