# Supplementary figures and images for: Screening and Application of DNA Markers for Novel Quality Consistency Evaluation in Panax ginseng
Source: Int J Mol Sci. 2025 Mar 17;26(6):2701. doi: 10.3390/ijms26062701 (PMC11942579; doi:10.3390/ijms26062701)

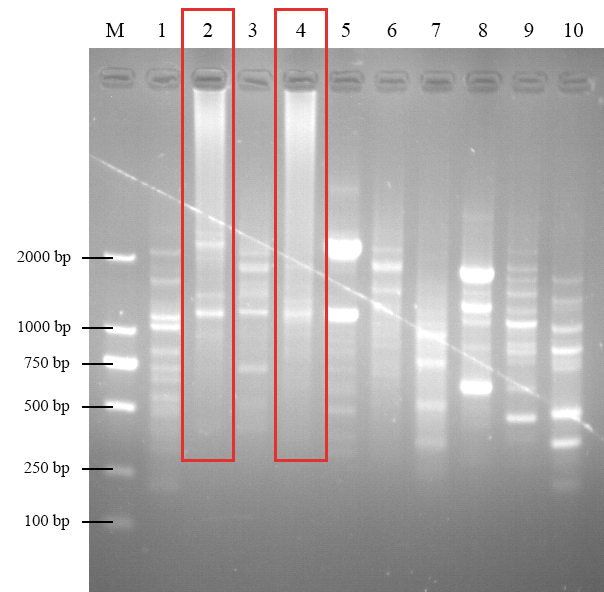

Supplement: Supplementary file 1 [file ijms-26-02701-s001.zip › Figure S1. Amplification results of initially selected 10 primers.tif]
